# The impact of achievement goals on college students’ English performance—A moderated mediation model

**DOI:** 10.1371/journal.pone.0310817

**Published:** 2025-01-07

**Authors:** Shaojie Tan, Arshad Abd Samad, Lilliati Ismail

**Affiliations:** 1 School of Foreign Languages, HuangShan University, Huangshan City, Anhui, China; 2 School of Education, Taylor’s University, Subang Jaya, Selangor, Malaysia; 3 Faculty of Educational Studies, Universiti Putra Malaysia, Seri Kembangan, Selangor, Malaysia; The Open University of Israel, ISRAEL

## Abstract

The study investigated the relationship between learning engagement and achievement goals, and English performance among college students. With the increasing popularity of online teaching methods, exploring how different teaching modes (online and classroom teaching) might influence students’ learning outcomes is important. The researcher sought to understand how adopting different achievement goals such as mastery and performance-avoidance approaches could impact English performance and learning engagement. By examining this factor, the study aimed to provide insights into effective teaching strategies and interventions that could enhance students’ academic success in English language learning. The survey included 953 college students assessed using the Achievement Goal Questionnaire and Learning Engagement Scale. Their IELTS English scores were also recorded to study the relationship between learning engagement and achievement goals, and English performance. Additionally, the researcher utilized statistical analysis tools such as SPSS and the PROCESS Marco programme to explore the moderated mediation model and to uncover the complex relationships among the variables in the study. The results revealed that adopting a mastery approach positively influenced English performance, while the performance-avoidance approach negatively influenced English performance. Additionally, learning engagement partially mediated the connection between the mastery approach, performance-avoidance approach, and English performance. Teaching mode influenced the initial phase of the mediating effect between the mastery approach and English performance. More importantly, compared to online teaching, classroom teaching with a focus on mastery approach had a stronger predictive effect on learning engagement. Lastly, there was a moderated mediating effect between English achievement and the mastery approach, whereas the performance-avoidance approach showed a simple mediating effect on English achievement. The findings from this research could potentially inform educators and policymakers on how to optimize teaching practices to promote student engagement and improve English language proficiency.

## Introduction

The English language serves as a vessel of knowledge and a repository of wisdom. Learning English is crucial not only for enhancing communication skills and academic standards but also for extensive global integration and personal development. English scores serve as vital indicators for measuring students’ academic performance, with numerous influential factors falling into two broad categories. The first category comprises personal factors, such as learning attitude, motivation, and anxiety. The second category encompasses environmental factors, including the teacher-student relationship, parental involvement, and family background [1]. Among the influential factors, achievement goals, learning engagement, and teaching modes emerge as significant contributors affecting English performance. Therefore, the researcher aimed to investigate the relationship between achievement goals and English performance while concurrently examining the mediating role of learning engagement and the moderating role of teaching modes. Through a systematic and comprehensive exploration, the aim was to uncover the underlying mechanism between achievement goals and English performance. By doing so, this research endeavors to provide targeted insights tailored to college students with diverse achievement goals.

The achievement goal theory is a motivation theory proposed by Dweck [2] from a social-cognitive perspective. It integrates ability beliefs, success or failure attributions, and emotions into the purpose of individual achievement behaviour as according to Dweck [2], different achievement goals have different impacts on academic performance. The impact of the mastery approach and performance-avoidance approach on academic performance has a relatively unified conclusion i.e. the mastery approach is significantly positively correlated with academic performance, and thus, it can positively predict academic performance. Conversely, performance avoidance is significantly negatively related to academic performance, and thus, it can negatively predict academic performance. However, research on mastery avoidance, performance approach, and academic performance shows inconsistent results. While most studies suggest that these factors are related, the correlations are not significant, particularly with regard to English achievement. Conversely, other studies suggest different findings. Baranik et al. [3] found that mastery avoidance is significantly negatively correlated with academic performance. Similarly, Chamorro and Furnham [4] believe that the performance approach has a significant negative correlation with academic performance. Based on this, Hypothesis 1 is proposed.

How do achievement goals affect college students’ English performance? Analyses of previous literature indicated that learning engagement is not only affected by achievement goals, but it can also predict English performance. At the same time, Roebken [5] found that achievement goals will affect academic performance by affecting learning engagement. Therefore, learning engagement is likely to be an important mediating variable between achievement goals and English scores. Schaufeli [6] believed that learning engagement is a state in which individuals maintain a fulfilling and focused spirit during the learning process and actively invest in learning. It is not only a specific indicator of the degree and intensity of learning involvement but also a common observation indicator of education quality and learning quality. Previous studies have confirmed that learning engagement is significantly positively correlated with academic performance and can positively predict academic performance, and negatively predict dropout rate. This is because students who invest more in learning can often flexibly use the mastery approach and effectively self-regulate, leading to naturally higher academic achievement [7]. The relationship between learning engagement and English performance is consistent with this. Learning engagement does not only predict the current English performance but also the academic performance in the next stage. On the other hand, students with different achievement goals also differ in the amount of time and energy they invest in learning. From existing research results, it can be seen that mastery approach, mastery avoidance, and achievement approach are significantly positively related to learning engagement and can directly predict learning engagement. When studying the relationship between achievement goals and English learning engagement, Liu et al. [8] also found that mastery approaches, mastery avoidance, and achievement approach do not only directly predict English learning engagement but also indirectly predict English learning engagement based on anxiety level towards English. Achievement avoidance, in contrast, shows a significant negative correlation with learning engagement and can directly predict learning negatively. Based on this, Hypothesis 2 is proposed.

Although achievement goals can directly or indirectly affect learning engagement, this effect is not static. Teacher leadership style can mediate the relationship between achievement motivation and learning engagement. Additionally, economic status plays an important role in learning motivation and learning engagement. Therefore, environmental factors may regulate the relationship between the two, and the teaching mode is the most important environmental factor in the learning process for students. Hence, it is important to examine how teaching methods affect achievement goals and learning engagement among college students. Teaching mode is a stable teaching activity structure framework established under the guidance of certain teaching theories and teaching ideas. It includes the traditional teaching mode as well as the new teaching mode. In the traditional teaching mode, teachers use traditional means such as blackboards, multimedia, and other teaching aids to teach teaching content in the classroom, while in the new teaching mode, teachers use online, hybrid, and flipped classroom teaching modes as well as other relevant modes. The researcher intends to compare whether there are differences in the relationship between college students’ achievement goals and learning engagement and classroom teaching and online teaching.

Online teaching breaks the limitations of time and space. It provides students with richer resources and teachers with opportunities to experience a variety of teaching methods. However, the openness, complexity, and virtuality of online teaching can easily lead to a lack of motivation to learn, lack of engagement in learning, poor emotional adjustment, inattention and other problems among students, which will ultimately lead to a negative impact on learning. Previous studies have shown that the learning environment (classroom or online teaching) affects learning motivation and that interactive factors in the learning environment can often positively predict learning motivation [9, 10]. To explore the current situation of learning motivation, Zeng and Guo [11] conducted an online teaching experiment on "Modern Educational Technology", and the results showed that the network environment, to a certain extent, has improved students’ learning motivation. However, Allan et al. [12] believed that online teaching has reduced the motivation of international students in colleges and universities to learn Chinese. As for learning engagement, 25% was caused by individual characteristics, while 75% was caused by the learning environment [13]. This indicates that student learning engagement varies between classroom and online teaching environments.

### Hypothesis

Some studies have found that there is no significant difference in learning engagement between general education courses based on SPOC (small-scale restricted online courses) and classroom large-scale general education courses [14, 15]. Some studies have shown that personal factors have a greater impact on online learning engagement than environmental factors, among which learning motivation is the personal factor with the greatest explanatory power [16, 17]. In online learning, learning motivations such as implicit intellectual beliefs, motivation regulation, and task value can all affect learning engagement. Students with higher learning motivation exhibit greater enthusiasm, commitment, and willingness to invest in online learning. A study was also carried out to explore the relationship between achievement goals and online learning engagement, involving 308 college students as research subjects. It was found that mastery approach, mastery avoidance, and achievement approach can all be connected in a positive direction. In predicting online learning engagement, the performance-avoidance path negatively predicts online learning engagement [18, 19]. The researcher aimed to explore whether teaching modes can mediate the relationship between achievement goals and learning engagement and to further determine whether there are deficiencies in online teaching. The following are the three hypotheses and hypothetical modes based on the above discussion:

H1: Different dimensions of achievement goals have an impact on English performance.
H1a. The mastery approach positively predicts English performance.
H1b. Performance avoidance negatively predicts English performance.
H1c. Mastery avoidance and performance approach are not significantly related to English performance.
H2: Learning engagement plays a mediating role between achievement goals and English performance.
H3: There is a moderated mediation model between college students’ achievement goals and English scores.

**Fig 1:**
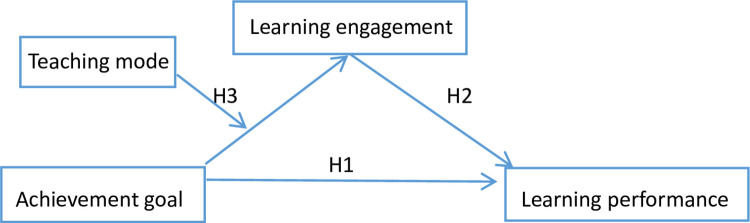
Moderated mediation model in achievement goal and learning performance.

Based on the assumptions, a moderated mediation model is proposed in Fig 1 to investigate this:

## Literature review

### Achievement goals

Achievement goals are fundamental in shaping individuals’ behaviours and outcomes within specific achievement contexts. Initially conceptualised as mastery and performance goals by Urdan and Kaplan [20], the framework has evolved into more nuanced categorizations, including mastery approach, mastery avoidance, performance approach, and performance-avoidance goals [21]. These goals guide individuals’ focus towards mastering skills, avoiding failure, achieving favourable outcomes, or avoiding unfavourable evaluations [22].

Elliot [23] proposed the hierarchical model of achievement motivation, emphasizing that achievement goals influence cognitive, emotional, and behavioral processes and outcomes. This model underscores that achievement goals are not merely task-oriented but are rooted in deeper psychological motivations and environmental influences.

### Learning engagement

Learning engagement encompasses behavioral, emotional, and cognitive dimensions [24]. Behavioral engagement refers to active participation and effort in learning tasks, while emotional engagement refers to positive or negative emotional experiences related to learning. Cognitive engagement involves the use of effective learning strategies and deep processing of information [25].

Halverson and Graham [26] define learning engagement as the integration of behavioral participation, cognitive inquiry, and positive emotional responses within the learning process. This multidimensional construct highlights the dynamic interaction between students’ behaviour, emotions, and cognitive strategies during learning activities.

### Relationship between achievement goals and learning engagement

The literature suggests a complex interplay between achievement goals and learning engagement. Achievement goals influence how individuals approach learning tasks, which in turn affects their engagement levels across behavioral, emotional, and cognitive domains.

Students pursuing mastery goals (both approach and avoidance) tend to exhibit higher levels of behavioral engagement by actively participating in learning activities and persisting in the face of challenges [27]. In contrast, those focused on performance goals may show variable engagement patterns depending on whether their goal is to achieve success or to avoid failure [28, 29].

Achievement goals also impact emotional experiences during learning. Mastery-oriented individuals often experience positive emotions such as interest and enjoyment, which contribute to sustained engagement and deeper learning [30]. Conversely, performance-oriented goals, especially avoidance-oriented ones, may lead to negative emotions such as anxiety or frustration, hindering emotional engagement [31].

Cognitive engagement involves the selection and application of effective learning strategies. Mastery-oriented goals encourage the use of deep cognitive strategies, fostering deeper understanding and integration of knowledge [32]. In contrast, performance-oriented goals may lead to superficial learning strategies aimed at achieving immediate success rather than deeper understanding [33].

Several factors influence the relationship between achievement goals and learning engagement. Positive teacher-student relationships and supportive classroom environments enhance students’ mastery goals and subsequently increase their engagement across behavioral, emotional, and cognitive domains [34, 35]. Peer relationships affect emotional engagement as students tend to engage more emotionally in learning activities when surrounded by supportive peers who share similar learning goals and investment levels [36]. The overall school environment, including size, culture, and disciplinary practices, can impact students’ achievement goals and subsequent engagement levels [37, 38].

Achievement goals significantly influence learning engagement across behavioral, emotional, and cognitive dimensions. Mastery-oriented goals generally lead to higher engagement levels by fostering positive emotional experiences, deeper cognitive processing, and sustained behavioral participation. In contrast, performance-oriented goals, particularly those focused on avoidance, may hinder engagement through heightened anxiety and less effective learning strategies. Future research could further explore the nuanced interactions between specific types of achievement goals and different facets of learning engagement within varied educational contexts.

### Learning engagement and academic performance

Studies have shown that learning engagement and academic performance are positively correlated [7, 39]. Moreira [40] conducted a study on 4,406 adolescent students from 68 schools in Portugal to explore the relationship between students’ learning support, learning engagement and academic performance. The results showed that compared to students with poor performance, students with better grades scored higher on cognitive engagement indicators and had higher scores on learning support. Xie et al. [41] conducted a study on 10,527 students in grades 9 to 12 in 20 public colleges in Ohio, USA, to explore their motivations. Results showed that social investment can significantly predict cognitive investment, and both social investment and cognitive investment are significantly positively related to academic performance.

In addition, a study of 298 graduate students was conducted to explore the role of student learning engagement and time perspective in academic performance [42]. The study found that students often demonstrate increased energy and engagement when confronted with academic challenges, leading to greater interest in learning activities, improved attention levels, and enhanced academic performance."

In summary, many foreign scholars have used different research subjects as samples to study the correlation between learning engagement and academic performance. The research results all show that there is a positive correlation between learning engagement and academic performance. On this basis, multi-variable correlation research provides strong support.

### Teaching modes

The impact of teaching modes on English performance is an area of research that has attracted much attention. Many researchers have explored this issue by comprehensively analysing the impact of online and classroom teaching modes on English performance.

Some studies focus on the online teaching mode and its advantages. For example, Kebritchi, Lipschuetz, and Santiague [43] found that an online teaching mode can provide opportunities for personalised learning, helping students learn at their own pace, and emphasised the positive impact of online interactive tools. Another study conducted by Means, Bakia, and Murphy [44] produced a comprehensive analysis which found that online education can achieve learning effects compared to traditional classrooms, and in some cases, achieve better results, indicating the positive impact of online teaching on English learning.

However, several studies have raised doubts about the limitations of the online teaching mode. For example, research by Lowes and Lin [45] found that students may face challenges when online teaching requires higher learning self-discipline and technical ability. Another study conducted by Tang, Hew, and Chen [46] showed that online teaching mode has less impact on students with lower autonomous learning willingness and self-regulation ability, and it has less impact on students with higher autonomous learning ability and self-regulation ability. The impact was more significant for students with adjustment skills.

Similar to online teaching, research on classroom teaching mode is also very important. Traditional face-to-face teaching can provide a more direct, interactive, and real-time learning experience that facilitates emotional connections and communication between teachers and students [47]. In addition, classroom teaching can also provide more cooperative learning opportunities, such as group discussions and team projects, which is considered to have a positive impact on English learning [48].

However, some studies have found that classroom teaching has some limitations. For example, classroom teaching may be limited by time and geography, and students may face problems such as transportation and accommodation [49]. In addition, some students may be unwilling to actively participate in classroom interactions due to shyness or low self-esteem. This shows that the impact of teaching mode on English performance is a complex issue. Online teaching mode can provide personalized learning and interactive tools, but it also presents challenges in learning self-discipline and technical ability; whereas, classroom teaching mode can provide real-time interaction and emotional connection but are limited by time and geography. Therefore, this article uses the teaching mode as a moderator variable to study how the relationship between achievement goals and academic performance works, and whether teaching mode has an impact on learning engagement.

## Methods

This study aimed to explore the relationship between achievement goals and learning engagement among college students, as well as to analyse the impact of different teaching methods (online versus classroom) on these variables. To achieve these objectives, a quantitative research approach was employed to analyse a 10-week teaching experiment conducted with the participants.

### Research design

The study utilized the purposive sampling technique to select 1,042 first-year students whose college entrance examination English scores ranged from 100 to 120. After excluding invalid questionnaires, 953 valid responses were collected, which is an effective recovery rate of 91.46%. The experiment was conducted from July 2022 till May 2023, during which students were randomly grouped into two types of learning: online and classroom. Before the commencement of the experiment, all students were provided informed consent and signed a consent form. Following the 10-week instruction period, students underwent an English Proficiency Test and completed two separate online questionnaires.

### Measures

#### Achievement goal questionnaire

The "Achievement Goal Questionnaire" used in the present study was derived from the work of Elliot and Murayama [50]. The questionnaire, encompassing a total of 29 items, was administered using a 5-point Likert scale, where a score of 1 denoted complete disagreement, and a score of 5 denoted complete agreement. The instrument targeted four distinct dimensions: Approach Mastery, Avoidance Mastery, Approach Performance, and Avoidance Performance. Higher scores on each dimension were indicative of more pronounced achievement goals. The internal consistency of the questionnaire was assessed using Cronbach’s alpha, yielding coefficients of 0.86, 0.88, 0.73, 0.83, and 0.85 for the total score and each of the aforementioned dimensions, respectively.

#### Learning engagement

The "Learning Engagement Scale" developed by Schaufeli et al. [6] was utilised in this study. This scale comprises a 5-point rating system (ranging from 1 for "never" to 5 for "always") and consists of three dimensions: vitality, dedication, and concentration, encompassing a total of 17 items. Individual scores reflect the level of investment in learning, where higher scores indicate greater engagement. The internal consistency of this scale, as measured by Cronbach’s alpha coefficient, was found to be 0.95 in the current study.

#### English performance

Regarding the measurement of English scores, standardization was ensured by employing a unified test paper across the participants. Specifically, the IELTS reading section was selected as the test measure which encompassed a total of five reading comprehension articles. Participants were tasked with completing 25 multiple-choice questions within a 50-minute time frame, with a maximum score of 100. To enhance reliability, two reviewers were involved in marking the test papers, with one teacher responsible for scoring and another teacher reviewing the results.

#### Data analysis

This study was conducted in two phases. In the first phase, 450 students were taught English online using the Chaoxing teaching platform, while 503 students continued to attend traditional classroom-based classes. The Chaoxing platform facilitated interaction between students and teachers, allowing for pre-class previews, in-class interactions, post-class reviews, etc. In a classroom setting, multimedia tools such as PowerPoint, audio, and video equipment were used to enhance teaching.

The ten-week course mainly focused on IELTS reading. The first stage of the course involved students taking a test in a classroom, which lasted 50 minutes. After completing the test, they proceeded to the second phase, which involved a questionnaire survey. Students were required to complete the questionnaire independently within 10 minutes The questionnaire data for the classroom teaching group were collected on-site. Then the performance data were collected and analyzed through the stage test using the unified IELTS reading test paper. SPSS 16.0 was used for descriptive statistics, correlation analysis, reliability analysis, and common method deviation test. The moderated mediation model was tested using the PROCESS macro programme.

## Results

### Confirmatory factor analysis

Before proceeding to structural equation modelling, this study first employed confirmatory factor analysis (CFA) to validate the factor structure of the scales. CFA was conducted for both the "Achievement Goal Questionnaire" and the "Learning Engagement Scale."

For the "Achievement Goal Questionnaire," a four-factor model was prespecified based on the theoretical constructs i.e. mastery approach, mastery avoidance, performance approach, and performance-avoidance approach. The CFA results revealed a Comparative Fit Index (CFI) of 0.95 and a Tucker-Lewis Index (TLI) of 0.94; both exceeding the 0.90 threshold. The Root Mean Square Error of Approximation (RMSEA) was 0.06, which is below the 0.08 criterion; and the Standardized Root Mean Square Residual (SRMR) was 0.05, also below the 0.08 cutoff. These indices indicated that the four-factor structure of the questionnaire has a good model fit.

A similar three-factor model was proposed for the Learning Engagement Scale, comprising vigour, dedication, and absorption. The CFA outcomes demonstrated a CFI of 0.96, a TLI of 0.95, an RMSEA of 0.07, and an SRMR of 0.06. These fit indices also confirmed the validity of the scale’s structure.

### Common method bias test

In studies involving common method bias testing, it is important to consider potential issues related to the source of data [51]. In this study, all variables were obtained from a single subject, which raises concerns about the common method bias. To address this issue, Harman’s single-factor analysis was employed to assess the presence of common method bias. The results revealed that seven out of all tested factors exhibited characteristic roots greater than 1. While this indicates the presence of multiple factors, it is noteworthy that the first factor only accounted for 30.625% of the total variance explained, which is below the critical threshold of 40%. These findings suggest no substantial evidence of significant common method bias within the study. Nonetheless, it remains important to consider and address any potential sources of bias to ensure the robustness of the results.

### Descriptive statistics and correlation analysis

Descriptive statistics and correlation analysis were employed to examine the four dimensions of achievement goals, learning investment, and English scores. Table 1 presents the results obtained. It was found that mastery avoidance and performance approach do not exhibit a significant relationship with English scores. However, the remaining variables show significant correlations with each other. Specifically, mastery approach is significantly positively correlated with mastery avoidance, achievement approach, learning engagement, and English performance. Moreover, mastery avoidance demonstrates a significant positive correlation with achievement approach, achievement avoidance, and learning engagement. Additionally, the performance approach also displays a significant positive correlation with performance avoidance and learning engagement; whereas, performance avoidance exhibits a significant negative correlation with learning engagement and English performance. Furthermore, a significant positive correlation is observed between learning engagement and English achievement.

**Table 1:** Descriptive statistics and correlation matrix of each variable (N = 953).

variable	M	SD	1	2	3	4	5
Mastery-approach	3.25	1.10					
Mastery-avoidance	3.03	1.12	.144**				
Performance-approach	3.09	1.13	.153**	-0.02			
Performance-avoidance	2.76	1.08	.203**	.127**	0.05		
Learning engagement	3.56	0.74	.275**	0.01	0.02	-.249**	
Online/classroom English score	70.93/71.10	1.16/1.16	.326**	-0.01	0.03	-.351**	.633**

*Note*: All correlations were significant at *P* < .01 (two-tailed testing of significance).

### Moderated mediation model test

Since the correlation between mastery avoidance, performance approach, and English performance is not statistically significant, the moderated mediation model involving these variables cannot be supported. Consequently, the analysis focused exclusively on examining the relationship between mastery approach, performance avoidance, and English achievement, disregarding the moderated mediation model.

Testing the mediating effect of learning engagement involved standardizing all data and implementing Mode 4 from the PROCESS module of the SPSS macro programme [52]. This test aimed to investigate whether learning investment operates as a mediator between achievement goals and English scores. Gender was included as a control variable. A mediation model was constructed with achievement goals as the predictor, learning investment as the mediator, and English achievement as the outcome variable. The Bootstrap method was employed with 5,000 repeated samples, and significance was determined based on whether the 95% confidence interval included zero.

Using the mastery approach as the predictor, Table 2 presents the path coefficients and their significance. Mastery approach significantly and positively predicted English scores (β = 0.33, t = 10.71, P<0.01), showing a significant total impact on English scores (Effect = 0.33, 95% CI [2.04, 2.96]). When accounting for the mediating variable, learning engagement and mastery approach continued to significantly and positively predict English performance (β = 0.17, t = 6.47, P<0.01). Moreover, mastery approach demonstrated a significant direct effect on English performance (Effect = 0.17, 95% CI [0.88, 1.65]), and it significantly and positively predicted learning investment (β = 0.28, t = 8.89, P<0.01). Additionally, learning investment significantly and positively predicted English performance (β = 0.59, t = 22.83, P<0.01), indicating a significant indirect effect of mastery approach on English performance (Effect = 0.162, 95% CI [0.116, 0.208]). This indirect effect accounted for 49.09% of the total effect, suggesting that learning investment partially mediates the relationship between mastery approach and English performance.

**Table 2:** Test of the mediating role of learning engagement.

predictor variable	Equation 1: English Score			Equation 2: Learning Investment			Equation 3: English Score		
	*β*	*t*	*95% CI*	*β*	*t*	*95% CI*	*β*	*t*	*95% CI*
mastery approach	0.33	10.71**	[2.04,2.96]	0.28	8.89**	[0.14,0.23]	0.17	6.47**	[0.88,1.65]
Learning investment							0.59	22.83**	[6.10,7.25]
gender	-0.04	-1.27	[-1.67,0.35]	-0.06	-1.91	[-0.18,0.00]	0.00	-0.17	[-0.88,0.74]
R2	0.11			0.08			0.43		
F	F(3,949) = 39.93, p = .000b			F(3,949) = .000b, p = .000b			F(4,948) = 176.72, p = .000b		
performance avoidance	-0.35	-11.61**	[-3.19,-2.27]	-0.25	-7.94**	[-0.21,-0.13]	-0.21	-8.28**	[-1.99,-1.23]
Learning engagement							0.58	23.08**	[6.05,7.17]
gender	-0.03	-0.97	[-1.49,0.51]	-0.05	-1.65	[-0.17,0.01]	0.00	0.03	[-0.79,0.81]
R2	0.13			0.07			0.44		
F	F(3,949) = 46.67, p = .000b			F(3,949) = .000b, p = .000b			F(4,948) = 187.85, p = .000b		

Note

*p< .05

**p< .001

Using performance avoidance as the predictor, Table 2 presents the path coefficients and their significance. Performance avoidance significantly and negatively predicted English performance (β = -0.35, t = -11.61, P<0.01), with a significant total effect (Effect = -0.35, 95% CI [-3.19, -2.27]). When incorporating learning investment as the mediating variable, performance avoidance continued to significantly and negatively predict English performance (β = -0.21, t = -8.28, P<0.01). Performance avoidance also exhibited a significant indirect effect on English performance (Effect = -0.21, 95% CI [-1.99, -1.23]). Furthermore, performance avoidance significantly and negatively predicted learning investment (β = -0.25, t = -7.94, P<0.001), while learning investment significantly and positively predicted English performance (β = 0.58, t = 23.08, P<0.01). This indicates a significant indirect effect of performance avoidance on English performance (Effect = -0.1254, 95% CI [-0.169, -0.082]), accounting for 35.8% of the total effect. Thus, these findings suggest that learning investment partially mediates the relationship between performance avoidance and English performance.

### Moderating effect of teaching mode

To investigate whether the relationship between achievement goals and English scores is mediated by learning engagement or by the teaching mode used in the first half of the course, a moderated mediation analysis was performed. All data were standardized, and Mode 7 of the SPSS PROCESS macro programme was employed for this analysis. Achievement goals were included as the predictor variables with learning engagement as the mediator, English scores as the outcome variable, and teaching mode as the moderator. A moderated mediation model was constructed, and significance was determined using the Bootstrap method with 5,000 repeated samples, assessing whether the 95% confidence interval included zero.

**Fig 2:**
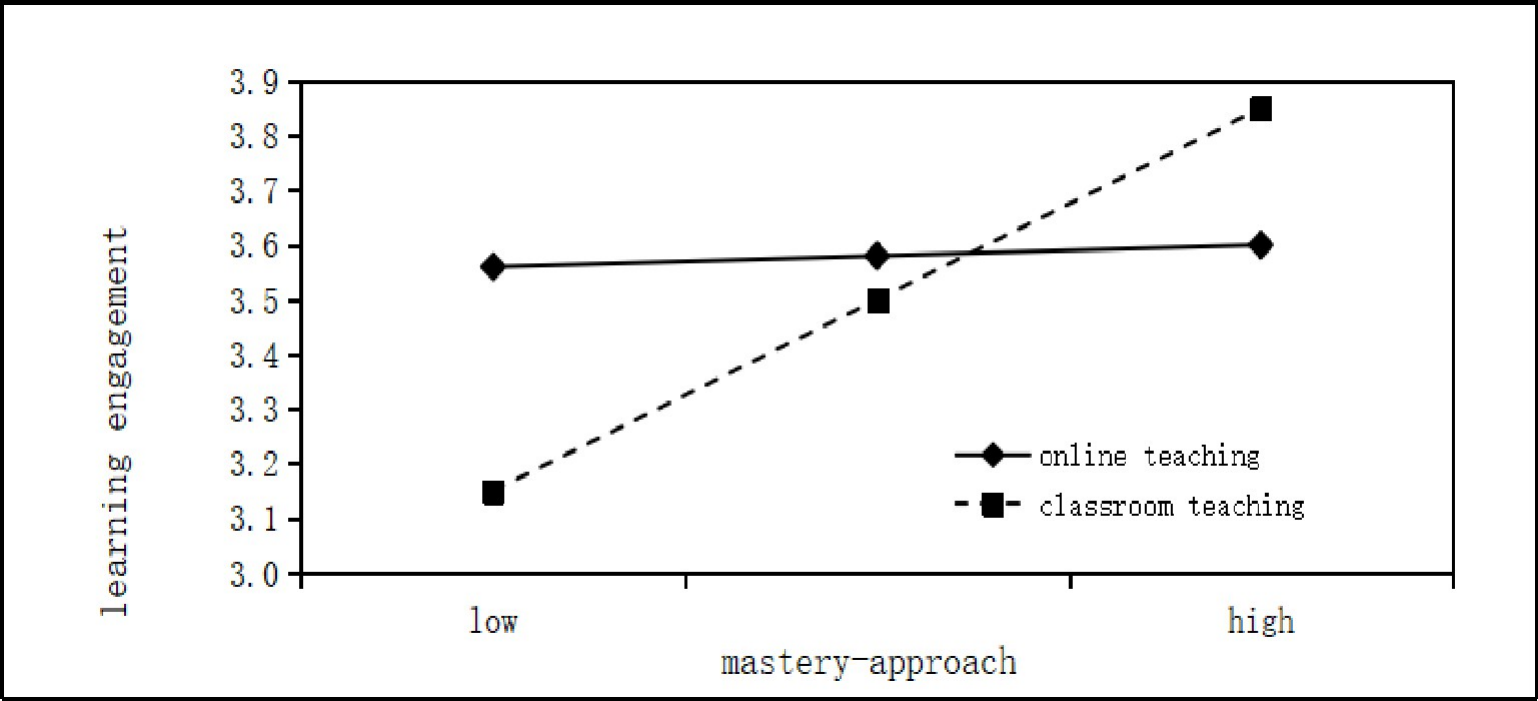
The moderating role of teaching mode between mastery approach and learning engagement.

The interaction between mastery approach and teaching mode significantly predicted learning engagement (β = 0.19, t = 6.38, P<0.01), indicating that teaching mode moderates the relationship between mastery approach and learning engagement. To further understand the moderating effect of teaching mode, separate simple slope analyses were conducted for online teaching and classroom teaching. The results of these analyses are presented in Fig 2.

In the context of online teaching, the positive predictive effect of mastery approach on learning engagement is not statistically significant (β = 0.021, t = 0.638, p = 0.524). However, in classroom teaching, the positive predictive effect of mastery approach on learning engagement is statistically significant (β = 0.319, t = 11.763, p<0.01). Further analyses revealed that in online teaching, the indirect effect of mastery approach on English performance through learning investment is 0.134 (95% CI [-0.383, 0.681]). On the other hand, in classroom teaching, the indirect effect of mastery approach on English performance through learning investment is 2.127 (95% CI [1.651, 2.622]). The index test results indicate a significant difference between the two indirect paths, with Index = 1.992 (95% CI [1.274, 2.726]), excluding zero. Consequently, it can be concluded that the teaching mode moderates the mediating effect of learning engagement on the relationship between mastery approach and English performance, specifically suggesting that college students with a mastery approach exhibit higher levels of learning investment and subsequently achieve better English scores in classroom teaching compared to online teaching.

Next, performance avoidance is utilised as a predictor variable. The path coefficients and their significance are presented in Table 3.

**Table 3:** Moderated mediation model between achievement goals and English performance.

predictor variable	Equation 1: Learning Engagement			Equation 2: English Score		
	*β*	*t*	*95% CI*	*β*	*t*	*95% CI*
mastery approach	0.33	10.82**	[2.05,2.96]	0.17	6.47**	[0.88,1.65]
Learning engagement				0.59	22.83**	[6.10,7.25]
Teaching mode	-0.03	-1.07	[-1.56,0.46]			
MA*TM	0.19	6.38**	[2.07,3.91]			
gender	-0.04	-1.25	[-1.62,0.36]	0.00	-0.17	[-0.88,0.74]
R2	.146			0.43		
F	F(5,947) = 33.49, p = .000b			F(4,948) = 176.72, p = .000b		
performance avoidance	-0.35	-11.38**	[-3.15,-2.22]	-0.21	-8.28**	[-1.99,-1.23]
Learning engagement				0.58	23.08**	[6.05,7.17]
Teaching mode	0.00	0.13	[-0.94,1.07]			
Mastery approach* teaching mode	-0.06	-1.82	[-1.82,0.07]			
gender	-0.03	-0.94	[-1.48,0.52]	0.00	0.03	[-0.79,0.81]
R2	.127			0.44		
F	F(5,947) = 28.71, p = .000b			F(4,948) = 187.85, p = .000b		

Note

*p< .05

**p< .001

The interaction term between performance avoidance and teaching mode does not significantly predict learning engagement (β = -0.06, t = -1.82, p>0.05). In terms of online teaching, the indirect effect value of performance avoidance on English performance through learning engagement is -0.658 (95% CI [-1.201, -0.143]), indicating a significant role of learning engagement in influencing performance avoidance and English performance under the online teaching mode. Similarly, in classroom teaching, the indirect effect value of performance avoidance on English performance through learning engagement is -1.148 (95% CI [-1.617, -0.676]), suggesting a similar mediating role of learning engagement between performance avoidance and English performance in the classroom setting. In summary, the teaching mode does not moderate the first half of the mediating pathway between performance avoidance and English performance via learning engagement. Consequently, the indirect effect of performance avoidance on learning engagement remains consistent across both online teaching and classroom teaching modes, indicating that teaching mode does not moderate the relationship between performance avoidance and learning engagement.

## Discussion

### Achievement goals and English performance

This study found that mastery avoidance and performance approach play significant roles in defining ability and its valence. However, as Urdan [20] suggested, these dimensions can have contrasting effects on English performance, potentially canceling each other out and resulting in no significant correlation with English performance. This intriguing finding prompts a deeper analysis within the framework of grand theories, such as the achievement goal theory which suggests that different achievement goals are associated with distinct patterns of learning outcomes [53].

This research reinforces the argument that achievement goals profoundly influence English performance, while also revealing that the interplay between mastery avoidance and performance approach can be complex [54]. While some students might be motivated by performance metrics, others may be hindered by a fear of not meeting expectations [55]. This insight is crucial for educators aiming to enhance college students’ English proficiency. Recognizing the importance of tailoring educational support to students’ individual achievement goals, educators should acknowledge the diversity of student aspirations and adjust their teaching methods accordingly [56].

For students focused on mastery-approach goals, reinforcing their learning paradigms and self-worth is paramount as it helps sustain their positive attitude towards learning [57]. On the other hand, students with a mastery-avoidance orientation require guidance to uncover their latent potential and to be exposed to a wider array of opportunities that can elevate their achievement aspirations [58].

Moreover, it becomes imperative to guide students—especially those with similar grades—towards understanding the true essence of learning, which extends beyond mere performance metrics to encompass ability development [59]. This shift in perspective could encourage students who initially exhibit mastery avoidance or performance approach tendencies to adopt a mastery orientation, aligning more closely with constructivist theories that emphasize depth of knowledge over superficial performance [60].

Lastly, for students with a performance-avoidance orientation, fostering resilience and recognizing their diligence can inspire them to confront and overcome avoidance tendencies, potentially leading to more substantial success in their English studies. Such an approach not only acknowledges the complexity of achievement motivations but also advocates for a nuanced pedagogical response that resonates with both expectancy-value and self-determination theories, emphasising the importance of perceived competence and autonomy in learning.

### Mediating role of learning engagement

The study reveals that both mastery approach and performance avoidance indirectly affect college students’ English performance through learning engagement. Specifically, college students who adopt a mastery approach tend to invest more in their learning, resulting in higher English scores. Conversely, those who exhibit performance avoidance tend to invest less in learning, leading to lower English scores, and this confirms Hypothesis 2. Previous research has demonstrated that study investment mediates the relationship between perseverance personality and academic performance, emphasizing the indirect influence of conscientiousness on academic performance via study investment [37]. Likewise, learning engagement serves as a mediating factor between gratitude and academic performance. These findings highlight the importance of learning engagement as a mediator between personality factors and academic performance. Moreover, some studies suggest that the intensity of learning motivation directly impacts the level of learning investment, which in turn affects academic outcomes. For instance, Tella [61] found that learning motivation not only directly influences academic performance but also indirectly affects learning investment, based on research involving 338 primary school students. Additionally, achievement goals have been found to predict learning investment, which subsequently impacts English performance, emphasizing the crucial role of learning engagement in connecting achievement goals and English performance.

Achievement goals have a direct impact on English performance and can also indirectly influence it through the mediating variable of learning investment. Therefore, increasing learning investment is an important measure for improving English performance. The definitions of learning investment vary slightly but generally encompass three aspects according to Fredricks et al. [62]. First, cognitive investment refers to the degree of commitment to the learning process, influenced by factors such as self-efficacy and learning motivation. Second, emotional investment relates to students’ responses to the external environment, with a focus on feelings of belonging and identity, largely influenced by academic emotions. Finally, behavioral investment encompasses interaction and participation both inside and outside the classroom, influenced by factors such as study persistence and time management.

To enhance English learning, English teachers should design questions of varying difficulty levels to match students’ abilities, ensuring that all students have opportunities to experience success. When students encounter challenging problems and struggle to solve them, timely comfort and encouragement should be provided to increase their self-efficacy and deepen their understanding of English, thereby fostering intellectual investment. Class teachers and psychology teachers should collaborate to incorporate emotional regulation strategies into class meetings and psychology classes. This approach can help students manage their academic emotions, allowing them to adjust their emotions promptly when facing difficulties and re-engage in English learning. Additionally, popularizing time management methods can enhance students’ time management skills and increase their behavioral investment. This will enable students to plan their study and daily routines more efficiently, ultimately improving their English learning outcomes.

### Moderating effect of teaching mode

The study revealed that in comparison to online teaching, college students who adopt a mastery approach in a classroom setting exhibit a stronger predictive effect on learning engagement [63]. This implies that students who have attained the mastery approach are more likely to invest in their learning during classroom-based sessions, leading to higher English scores, thus corroborating Hypothesis 3. Specifically, mastery orientation demonstrates a positive and significant correlation with deep processing strategies, whereas it exhibits a negative correlation with effort avoidance and self-worth protection strategies. Students with mastery approach tend to employ various forms of self-regulated learning strategies and engage in supportive online learning behaviors to enhance their learning experience, while those with a performance-avoidance mindset do the opposite. While college students with a mastery approach actively resist the drawbacks associated with online teaching, they are still influenced by these challenges to a certain degree. Consequently, the mediating role of learning engagement becomes more prominent in classroom teaching [64]. On the other hand, students with a performance-avoidance orientation are inherently fearful of under-performing in comparison to their peers and seek to evade negative evaluations of their abilities. Therefore, whether in the context of online or classroom teaching, teaching modes have a limited impact on these students, as they consistently maintain a negative attitude towards learning.

In light of this, a hybrid teaching approach could be adopted for future English instructions, with classroom teaching as the primary mode, supplemented by online teaching [65]. This approach aims to leverage the advantages of online teaching while mitigating the limitations of in-person instruction. However, the efficacy of online teaching depends on meeting certain conditions. Firstly, a unified teaching platform should be established to provide shared resources for online courses, allowing equal learning opportunities for all students. Secondly, a professional team of teachers is required for online instruction, with higher expectations in terms of solid instructional design capabilities, advanced information literacy, and a firm belief in technical teaching. Thirdly, it is crucial to design engaging and stimulating learning activities, as learning motivation enhances student participation and yields better learning outcomes. Therefore, online teaching should emphasize the incorporation of exploratory, authentic, collaborative, and technically supported learning activities. Fourthly, the selection of teaching content should be strategic, targeted, and diverse. Not all English concepts are suitable for online teaching; hence, content that maximizes the advantages of online instruction should be chosen to ensure meaningful learning experiences. Additionally, considering the varying knowledge gaps among students, online teaching should be tailored to their individual abilities, offering suitable content at different proficiency levels through teaching videos. Finally, online teaching should adopt diverse formats, encompassing recorded videos, live broadcasts, teacher presentations, and real-time demonstrations, rather than being restricted to a single modality.

## Conclusion

In revisiting the objectives of this study, it sought to scrutinise the intricate relationships between achievement goals, learning engagement, teaching modalities, and their collective impact on university students’ English language proficiency. The empirical findings of this research have illuminated that a mastery orientation significantly enhances English performance, while a performance-avoidance orientation is associated with a decline in proficiency. These insights are pivotal for educators and instructional designers aiming to cultivate learning environments that accentuate mastery-oriented goals, thereby catalyzing superior English performance among students.

Furthermore, the study elucidates the central role of learning engagement as a mediator in the relationship between achievement goals and English performance. High levels of engagement serve to amplify the beneficial effects of a mastery orientation, whereas reduced engagement exacerbates the detrimental impacts of performance avoidance. This observation underscores the need for educators to engender deeply engaged learning atmospheres, which are critical for optimizing educational outcomes, specifically within the domain of language acquisition.

Additionally, our analysis reveals that teaching modality acts as a moderator, with traditional classroom instruction bolstering the positive relationship between mastery orientation and learning engagement, in contrast to online teaching methods. This finding accentuates the importance for educators to capitalize on the distinctive strengths of classroom environments to elevate student engagement and promote effective language learning.

This investigation contributes to the existing literature on achievement goal theory by delineating the mechanisms through which these constructs influence English performance. From a practical standpoint, the insights garnered from this research offer invaluable guidance for educators and policymakers on enhancing English language education. By customizing pedagogical approaches to nurture mastery orientations and mitigate performance avoidance tendencies, educational institutions can facilitate more effective learning experiences and enhance students’ overall linguistic competencies.

Notwithstanding the contributions of this study, I acknowledge several limitations that warrant consideration. The reliance on questionnaire data necessitates the need for qualitative methodologies, such as interviews and observational studies, to complement quantitative survey instruments. This integrated approach would yield a more comprehensive understanding of the phenomena under examination. Moreover, the homogeneity of our sample, sourced exclusively from a single academic institution, constrains the generalisability of our findings. Future research should endeavour to broaden the demographic scope by including participants from diverse geographical locations and academic levels to fortify the external validity of the conclusions.

Furthermore, the recommendations offered herein remain speculative, advocating for interventions that align with our empirical findings but lack empirical validation of their efficacy. Future studies are recommended to adopt experimental designs that rigorously test the effectiveness of proposed interventions designed to modify achievement goals and learning engagement, with the ultimate objective of corroborating their impact on English language performance.

In sum, this research has elucidated the multifaceted interconnections between achievement goals, learning engagement, teaching modalities, and English language proficiency among university students. This extends an invitation to the scholarly community to further probe these domains to refine educational practices and policies, ensuring they are anchored in robust empirical evidence, thus contributing to the enhancement of language education standards.
